# Supplementary material for: Impact of Wuhan lockdown on the indications of cesarean delivery and newborn weights during the epidemic period of COVID-19
Source: PLoS One. 2020 Aug 13;15(8):e0237420. doi: 10.1371/journal.pone.0237420 (PMC7425855; doi:10.1371/journal.pone.0237420)
Supplement: Attachment — Submitted filename: Response to Reviewers.docx [file pone.0237420.s002.docx]

Dear Editors and Reviewers:

Thank you very much for reviewing our manuscript with the **ID: PONE-D-20-10095**.

We have revised our paper following your comments. Our paper entitled “**The Influence of lockdown on the indications of cesarean delivery and newborn weights during the epidemic period of COVID-19 in Wuhan**”, which we wish to be considered for publication in “**PLOS ONE**”.

We highly appreciate the reviewers’ and editors’ carefulness, conscientious, and the broad knowledge on the relevant research fields, since they have given us a number of beneficial suggestions. We also appreciate the fact that the reviewer and editor find merit in the research and are willing to consider a new submission for publication. We have read the comments carefully and have made all corrections corresponding to reviewers’ criticism and suggestions which we hope to meet with your approval.

We have considered carefully on the comments of the reviewer for the sincerely. Here, we submit a carefully revised version that addresses all the concerns. In the revised manuscript, we highlight the major changes using blue coloured text.

We provide a detailed response to the reviewers. All the concerns are grouped and highlighted in the form of italics and blue, and following that are the corresponding responses.

**Response to Editor**

*1. There is currently a great deal of interest on the short and long-term health effects of COVID-19, including on unborn babies. The title of the manuscript suggests that the current work provides some enlightenment in this area. However, as pointed out by all Reviewers, the title is greatly misleading as only a few of the pregnant women in this cohort were COVID-19 positive, which precludes any interpretations on how this disease may affect their unborn babies. Instead, the main subject matter of this work is how does quarantine of late-stage pregnant mothers affect initial health outcomes in their babies relative to a similar cohort (although there are some seasonal differences) of pregnant mothers not subjected to such quarantine. As noted by Reviewer 1, the paper provides some comfort to many pregnant women who are currently having to remain primarily at home that such quarantine will likely not dramatically affect their babies, although maternal physical inactivity due to the quarantine may lead to heavier babies.*

**Response:** Thanks for your comments. To address the misleading problem, we have revised the manuscript title as “The influence of Wuhan lockdown on the indications of cesarean delivery and newborn weights during the epidemic period of COVID-19”.

The key findings of our manuscript lie in three aspects:

Firstly, as noted by Reviewer 1, the paper provides some comfort to many pregnant women who are currently having to remain primarily at home that such quarantine will likely not dramatically affect pregnancy complications. The difference on most of the indications of cesarean delivery is very small between the observation group and control group.

Secondly, we show that the cesarean delivery on maternal request (CDMR) and fetal distress also increased significantly in the observation group. As reviewer 2 pointed out, this is interesting, and could be from women postponing deliveries during the pandemic. Increases in maternal stress due to the pandemic and all of the associated health fears may also be a factor.

Thirdly, there was a slight increase in offspring weights for full term or close to full term births. Based on this finding, we suggest that pregnant women should adjust their diet structure and take appropriate exercises to reduce the potential risks associated with heavy offspring weights.

In addition, we have revised the whole manuscript following the research topic of “influence of lockdown on the indications of cesarean delivery and newborn weights” **rather than** “the pregnancy outcomes of women who had been infected with COVID-19”.

*2. I agree with Reviewers 2 and 3 that the manuscript needs to be heavily edited to improve its clarity, and an individual with statistical expertise is likely needed to address some of the statistical concerns raised.*

**Response:** In the revised process, a professor in Huazhong University of Science and Technology with rich statistical expertise have helped us to address the statistical concerns raised. The Reviewer 2 raised a comment that the procedure of the PSM method used to generate the control group should be introduced. We added this procedure as “*Actually, three basic variables of age, gravida, and para were used to automatically select 7159 pregnant women in the SPSS software as the control group from the 29799 data before the lockdown using the PSM method. The balancing score of the two groups were 0.10312 and 0.10590, which were very similar.”*  The process is presented in the following two figures.

As shown in Table 2 of the revised manuscript, the difference on the caesarian delivery (CD) rate between the observation group and the control group is not statistically significant (*p>0.05*). As noted by Reviewer 2, if a difference is not statistically significant, there is no difference. In the revised manuscript, we have corrected this incorrect conclusion in the sections of abstract, results, and discussions. We also removed all the explanations on the slight increase of CD rate in the discussion section.

*3.* *Based on the comments raised by all three Reviewers, I am rejecting the current paper. However, I would like to encourage the authors to submit a new paper based on one of two approaches. The first approach would involve altering the title and general paper to reflect the actual studies and that avoids any claims or misinterpretations that the work examines health effects of maternal COVID-19 infection on unborn babies. The title and claims of the paper has to make clear that the primary extrinsic factor studied is how maternal quarantine affects newborn health. The second option, which is preferable, is to include additional offspring data from Coronavirus + mothers such that these results can be compared to infants born to mothers without this disease. Even such a pilot study would likely provide meaningful results whether differences are obtained or not. I hope that the investigators have such data available to them that they can proceed with this option that will assuredly have higher impact.*

**Response:** Thanks for providing the two approaches for revising the manuscript. Following our research design and your valuable comments, we adopt the first approach suggested to revise the whole paper. We agree with you that the research problem that the health effects of maternal COVID-19 infection on unborn babies deserves extensive attention. However, including additional offspring data from Coronavirus+ mothers is highly difficult as the cases of pregnant women with confirmed COVID-19 infection are limited, which will inevitably affect the statistical reliability. Actually, our manuscript is focused on the impact of lockdown on indications of cesarean delivery and newborn weights, which can be seen from the collection or selection of observation group and control group. Based on the first approach, we have revised the title and removed all the misleading sentences.

*\*

**Response to Comments of Reviewer 1**

*1. This is a timely paper, although a reader might mistakenly believe that the topic has to do with the pregnancy outcomes of women who had been infected with CORVID19, whereas it actually compares an extensive cohort of women, mostly uninfected, who gave birth during the lockdown in Wuhan between January 23 to March 24 2020, and a matching group from a three-week period of January from the previous year.*

**Response:** Thanks for pointing out the problem. Following your comments, we have revised the tile to avoid misleading. Our manuscript is focused on the indications of cesarean delivery and newborn weights during the epidemic period of COVID-19 in Wuhan.

*2. In fact the number of women positive for CORVID19 was too small to provide statistically meaningful data. It should also be noted that all these women must have become infected* *in the third* *trimester* of their pregnancies*. It will be another six months or so before we will know whether infections early in pregnancy caused serious problems, such as developmental effects or fetal loss (as noted, for example, with Zika virus infections).*

**Response:** We fully agree with you the that the pregnant women positive for COVID-19 who were affected in any stage of the three trimesters of their pregnancies might lead to serious problems, such as developmental effects or fetal loss. It brings a lot of research problems deserving extensive future attention. However, as emphasized in our manuscript, we focused on the influence of lockdown on the indications of cesarean delivery and newborn weights during the epidemic period of COVID-19 in Wuhan rather than the negative outcome of pregnant women with COVID-19 infection.

*3.* *Importantly, the data show that the lockdown was not correlated with serious pregnancy complications.* *There was a slight increase in offspring weights for full term or close to full term births and a slight increase in the number of elected cesarean deliveries compared to controls. Although these minor changes were attributed to the lockdown, it should be emphasized by the authors that the control group was from a short period in January the previous year. Climate, food type and consumption and other non-controlled inputs in that narrower period of time might have affected the data in this relatively poorly controlled study in which differences were quite small.*

**Response:** We highly appreciate your valuable comments on our findings. In the discussion section of the revised manuscript, we have included “ *the findings might be affected by other factors, such as climate, food type, and other non-controlled inputs, due to the fact that the* *observation group was from a short period in 2020. The research question investigated in this study needs further exploration with other data sets.***”**

*4. A shortening of the manuscript, particularly the speculative Discussion is warranted.*

**Response:** Thanks for this suggestion. We have shortened the manuscript by removing some sentences or compressing some paragraphs. In the discussion section, we removed all the possible explanations on the slight increase of CD rate, because, followed by the two reviewers’ comments, the difference between the observation group and control group was not statistically significant. In addition, we also removed the discussions on the possible effect of COVID-19 by reviewing the literature with respect to Asian flu epidemic and SARS, because we focus on the impact of lockdown on the indications of cesarean delivery and newborn weights during the epidemic period of COVID-19 in Wuhan.

*5. Although the quality of the written English is generally good, there are areas where improvement is needed. For example on line 55 (Abstract) it is stated that that the increased weight of the newborns is due to "transportation difficulties", whatever that means. Not only is this sentence difficult to understand, there is really no clear explanation for increased weight gain, although lack of exercise by the mothers could be a contributory factor.*

**Response:** Thanks for your comments. In the revised process, we have carefully checked all the grammar mistakes and writing problems. In the abstract, we have revised the conclusion on offspring weights as “Furthermore, we find that there was an increase in offspring weights for full term or close to full term births (*p<0.05)*.” We also made the corresponding revisions on the discussion section (Lines 196-197) by adding “*Through comparison between the observation group and control group, we show that lockdown may lead to heavier babies for the newborns whose gestational age were no less than 34 weeks.*” To be clear and concise, we revised the explanations on the possible causes of increased weight gain of newborns: “*During pregnancy, balanced nutrition and sufficient vegetables and protein have a positive impact on the birth weight of the newborn [14-16]. However, affected by the epidemic of COVID-19 infection in Wuhan, supermarkets and vegetable markets were closed and rich food access was relatively restricted. Relative lack of vegetables and crude fiber foods, and the increased intake of foods with high level of carbohydrates such as rice and noodles, because they were easily obtained and stored, lead to the increase of the offspring weight. On the other hand, after the city was locked down, the activities and exercise of pregnant women was decreased significantly. We commend that in the situation of city lockdown due to COVID-19, pregnant women should try to satisfy the nutritional balance, increase the intake of protein and vegetables as much as possible, control the intake of carbohydrates such as rice noodles, and increase indoor activities.*”

**Response to Comments of Reviewer 2**

1. *The authors of this manuscript report a variety of parameters related to late pregnancy, Caesarian deliveries and various other metrics in the Wuhan, China region during the time that the pandemic swept through that area. The data is based on several thousand infants born during the lockdown that occurred in early 2020 compared to a somewhat larger group that was born in early 2019 and served as controls. The authors report that the number Caesarian deliveries did not rise. There were some minor changes noted in terms of weights of late term infants, and fetal distress.*
   **Response:** Thanks for your positive comments on the findings of our manuscript.
2. *Since the onset of the pandemic in Wuhan, there has been great interest in and concern over whether the infection of a pregnant mother would result in any changes in the fetus during late gestation or the infants after birth. The recent experience with the Zika virus, which induced abnormalities in the offspring, is something that clearly raises at least the concern that the coronavirus could have some effects on the offspring. The SARS virus was similar in that it also caused pregnancy complications, as the authors indicate and discuss, although these were dramatically different than with Zika. Unfortunately, the current study does not provide data to address this critical point. The question the authors do address, whether there were changes in caesarian deliveries and other metrics during the lockdown is perhaps of some value as well, although clearly the question on everyone’s mind all over the world is whether maternal infection with coronavirus affects the fetus/newborn and produces permanent abnormalities.*

**Response:** We highly appreciate your valuable suggestions and positive comments on the value of our manuscript. The infection of SARS-CoV-2 of pregnant women may pose a threat to both maternal and fetal safety, which deserves extensive attention. This study is focused on another important problem: how the lockdown policy affects the indications of cesarean delivery and newborn weights during the epidemic period of COVID-19 in Wuhan. In current situation, the life-threating COVID-2019 has rapidly spread across global countries and a lot of government agencies have implemented the lockdown policy. In contrast to the *Zika* and SARS virus, the Wuhan lockdown was an unanticipated action in responding the spreading of infectious diseases. This motivates us to explore the impact of the lockdown policy.

3. *While the difficulties of writing a manuscript in a language other than your native language are certainly acknowledged, the manuscript contains many instances where the English is subpar or just incorrect. There are numerous commercial services that help with writing scientific papers, and it is recommended that the authors consider use of this type of service.*

**Response:** In the revising process, we have carefully revised all the grammar mistakes and sentences to increase readability. In the revised manuscript, all the revisions are colored in blue. In addition, a professional institution has offered us the paid editing service. The certificate of editing is shown in the following figure.

4. *The authors used propensity score matching to compare their two groups. The authors need to describe how they selected the matching population according to additional covariables. They should also mention the balancing score. These items are critical for fully explaining the data, but in the manuscript these were not explained well enough.*

**Response:** Thanks for pointing out this important problem. We have added this procedure as “*Actually, three basic variables of age, gravida, and para were used to automatically select 7159 pregnant women in the SPSS software as the control group from the 29799 data before the lockdown using the PSM method. The balancing score of the two groups were 0.10312 and 0.10590, which were very similar.* ”The process is presented in the following two Figures.

5. *Line 145-147: it is mentioned that the overall Caesarian rate was increased, but this was not statistically significant. If a difference is not statistically significant, there is no difference. The authors need to correct this in their manuscript.*

**Response:** Thanks for pointing out this important problem. As *p*>0.05, the difference on the caesarian delivery (CD) rate between the observation group and the control group is not statistically significant. In the revised manuscript, we corrected this incorrect conclusion in the sections of abstract, results, and discussions. We also removed all the explanations on the slight increase of CD rate in the discussion section.

6. *Tables in a manuscript are supposed to stand alone. Thus, in Table 3, the authors need to define CDMR. There are two Table 4s; this problem should be corrected. In the second Table 4, the authors show the body weights of the newborns in the study. They show the weights down to 0.01 g. Obviously the newborns are not being accurately weighed down to 0.01 g. The authors should review the rules regarding significant digits, and correct this table accordingly.*

**Response:** Thank you very much. To stand alone, we have replaced the abbreviation name “CDMR” with the full name “Cesarean delivery on maternal request (CDMR)”. The second Table 4 has been numbered as “Table 5”. By reviewing the literature (Kitajima M, Oka S, Yasuhi I, et al. 2001; Pickerel K K, Waldrop J, Freeman E, et al., 2020; Duydu Y, Başaran N, Üstündağ A, et al. 2018), the decimal parts of the mean and standard deviation of the newborn weight are removed following the rounding rule. The decimals of other variables are also revised.

7. *The increases in Caesarian deliveries during the lockdown, when the women were apprehensive about staying in the hospital, is interesting but somewhat predictable.*

**Response:** Thanks for the comments. Actually, before this investigation, we also believed that the rate of Caesarian delivery will increase during the lockdown. However, this study obtained a counter-intuitive conclusion that the Caesarian delivery rate was not significantly related to the lockdown policy. This might be attributed by the fact that during the period, our hospital was identified as a non-designated hospital with fever clinics to accept normal pregnant women and suspected pregnant women. The suspected patients who were laboratory-confirmed COVID-19 infection were then transferred to designated hospitals for further treatment. The infection risk was controlled well.

8. *The authors show that fetal distress was increased in the infants born during the pandemic. This is interesting, and could be from women postponing deliveries during the pandemic. Increases in maternal stress due to the pandemic and all of the associated health fears may also be a factor in these data. Overall, a modest amount of new data are presented in this manuscript, but the manuscript also has a number of problems in terms of the data presentation and analysis and writing.*

**Response:** Thanks again for your positive comments. The problems or comments raised by the editors and reviewers are invaluable to the improvement of our manuscript and have been addressed carefully in the revised manuscript.
